# An uncommon etiological factor for aspiration pneumonitis caused by spontaneous sphenoid sinus meningoencephalocele with cerebrospinal fluid rhinorrhea: a case report

**DOI:** 10.1186/s12890-021-01620-5

**Published:** 2021-07-31

**Authors:** Jiayu Cao, Wei Liu, Li Wang, Yujuan Yang, Yu Zhang, Xicheng Song

**Affiliations:** 1 Department of Otolaryngology-Head and Neck Surgery, Yantai Yuhuangding Hospital, Qingdao University, Yantai, 264000 China; 2 Department of Physical Examination, Yantai Yuhuangding Hospital, Qingdao University, Yantai, China

**Keywords:** Aspiration pneumonitis, Meningoencephalocele, Sphenoid sinus, Etiology, Postnasal drip

## Abstract

**Background:**

Aspiration pneumonitis is an inflammatory disease of the lungs which is difficult to diagnose accurately. Large-volume aspiration of oropharyngeal or gastric contents is essential for the development of aspiration pneumonitis. The role of cerebrospinal fluid (CSF) rhinorrhea is often underestimated as a rare etiological factor for aspiration in the diagnosis process of aspiration pneumonitis.

**Case presentation:**

We present a case of a patient with 4 weeks of right-sided watery rhinorrhea accompanied by intermittent postnasal drip and dry cough as the main symptoms. Combined with clinical symptoms, imaging examination of the sinuses, and laboratory examination of nasal secretions, she was initially diagnosed as spontaneous sphenoid sinus meningoencephalocele with CSF rhinorrhea, and intraoperative endoscopic findings and postoperative pathology also confirmed this diagnosis. Her chest computed tomography showed multiple flocculent ground glass density shadows in both lungs on admission. The patient underwent endoscopic resection of meningoencephalocele and repair of skull base defect after she was ruled out of viral pneumonitis. Symptoms of rhinorrhea and dry cough disappeared, and pneumonitis was improved 1 week after surgery and cured 2 months after surgery. Persistent CSF rhinorrhea caused by spontaneous sphenoid sinus meningoencephalocele was eventually found to be a major etiology for aspiration pneumonitis although the absence of typical symptoms and well-defined risk factors for aspiration, such as dysphagia, impaired cough reflex and reflux diseases.

**Conclusions:**

We report a rare case of aspiration pneumonitis caused by spontaneous sphenoid sinus meningoencephalocele with CSF rhinorrhea, which can bring more attention and understanding to the uncommon etiology for aspiration, so as to make more accurate diagnosis of the disease and early surgical treatment.

## Background

Aspiration pneumonitis is an infectious disease of the lungs caused by large-volume aspiration of oropharyngeal or upper gastrointestinal contents, which is more likely to occur in patients with risk factors such as impaired swallowing, consciousness, and cough reflex, etc. [1]. However, aspiration pneumonitis is sometimes difficult to diagnose in the first place due to atypical clinical features and uncertain etiology. We described such a case of aspiration pneumonitis caused by spontaneous sphenoid sinus meningoencephalocele with cerebrospinal fluid (CSF) rhinorrhea.

## Case presentation

**Fig. 1:**
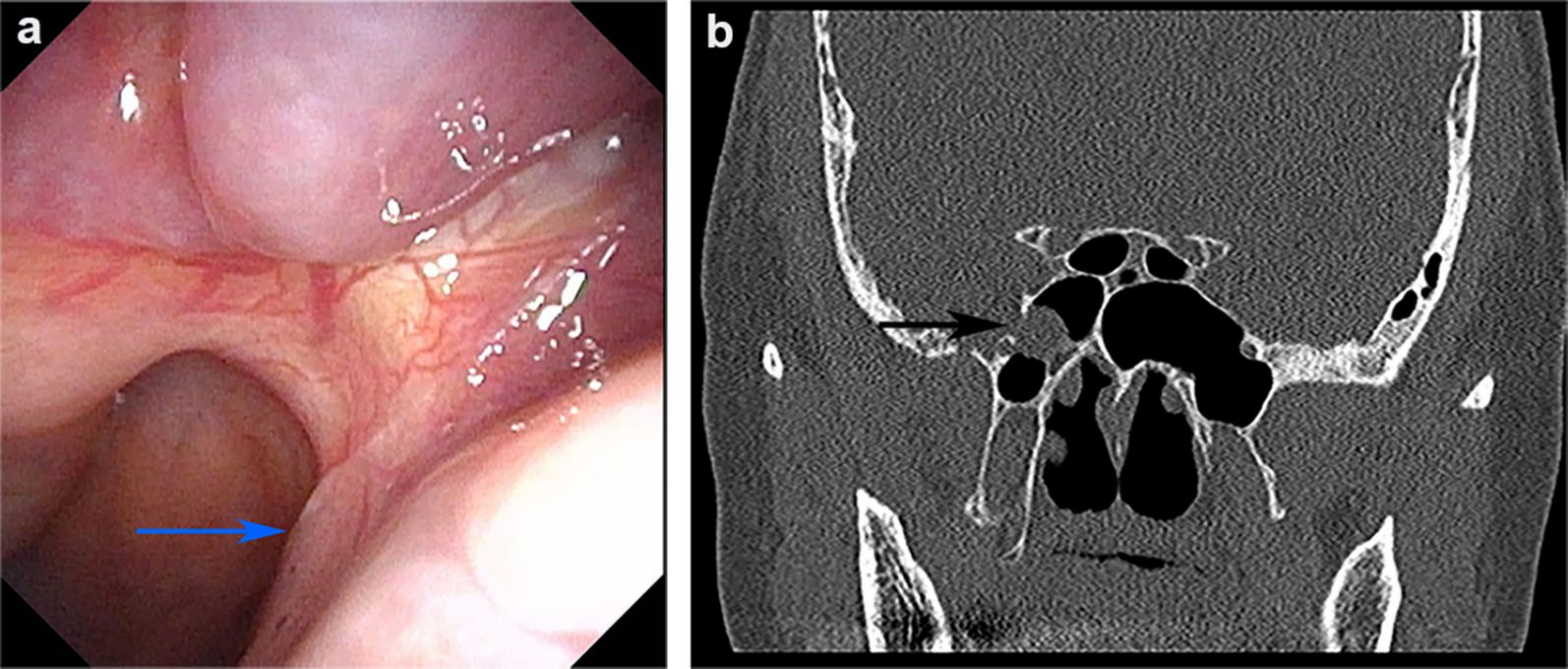
Preoperative endoscopy image and sinus computed tomography (CT) scan. **a** Preoperative endoscopy image. The blue arrow indicates the persistent flow of cerebrospinal fluid (CSF) backwards into the right posterior nostril. **b** Sinus CT scan. The black arrow indicates the place of lesion tissue protruding into right sphenoid sinus

A 62-year-old female presented with 4 weeks of right-sided watery rhinorrhea. She reported spontaneous clear, salty nasal drainage without a history of trauma. Two weeks later, she developed a frequent dry cough caused by postnasal drip and gradually worsened especially when lying down, which prevented her from falling asleep. The patient underwent nasal cavity examination and computed tomography (CT) scan of sinuses after visiting department of otolaryngology, and we found a continuous flow of clear fluid to posterior pharyngeal wall (Fig. 1a) and sinus CT showed the lesion tissue extending from the right middle cranial fossa into sphenoid sinus through a local bone defect in the lateral wall of the right sphenoid sinus (Fig. 1b). Combined with measurement of glucose level in nasal secretions (4.4 mmol/L), the patient was initially diagnosed with spontaneous sphenoid sinus meningoencephalocele accompanied by CSF rhinorrhea, and she was admitted to department of otolaryngology -head and neck surgery on June 29, 2020.

**Fig. 2:**
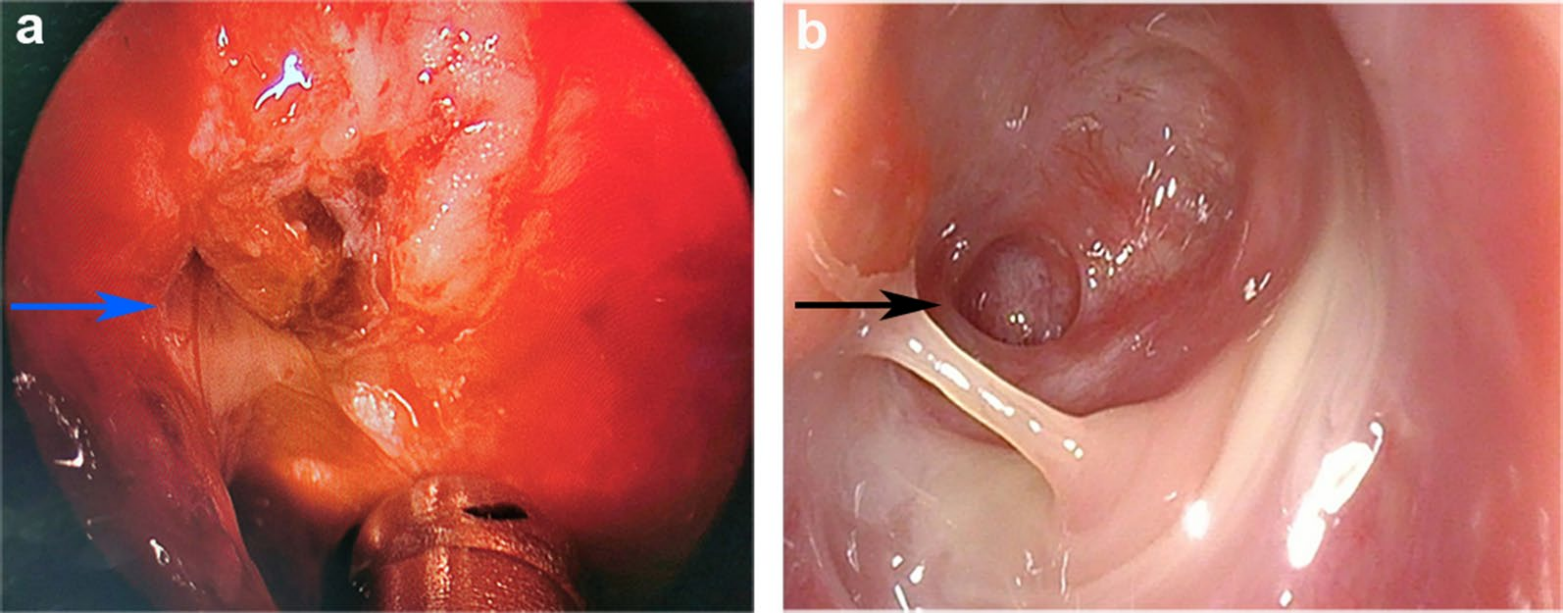
Endoscopy images. **a** Intraoperative endoscopy image. The blue arrow points to the lesion tissue and skull base defect in the lateral wall of the right sphenoid sinus. **b** Endoscopy image 2 months after surgery. The black arrow indicates repaired area of the skull base defect

**Fig. 3:**
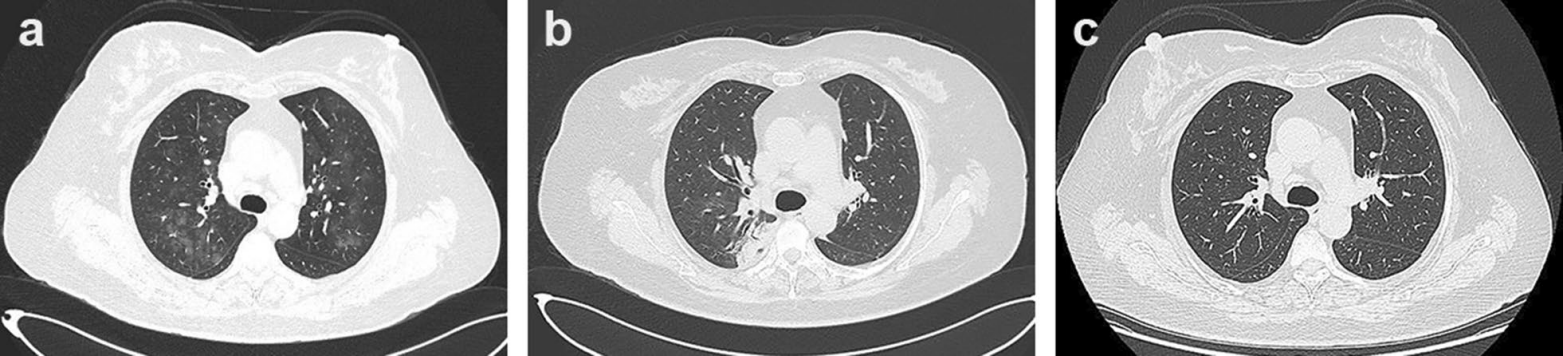
Chest CT images. **a** Preoperative chest CT image. **b** Chest CT image 1 week after surgery. **c** Chest CT image 2 months after surgery

**Fig. 4:**
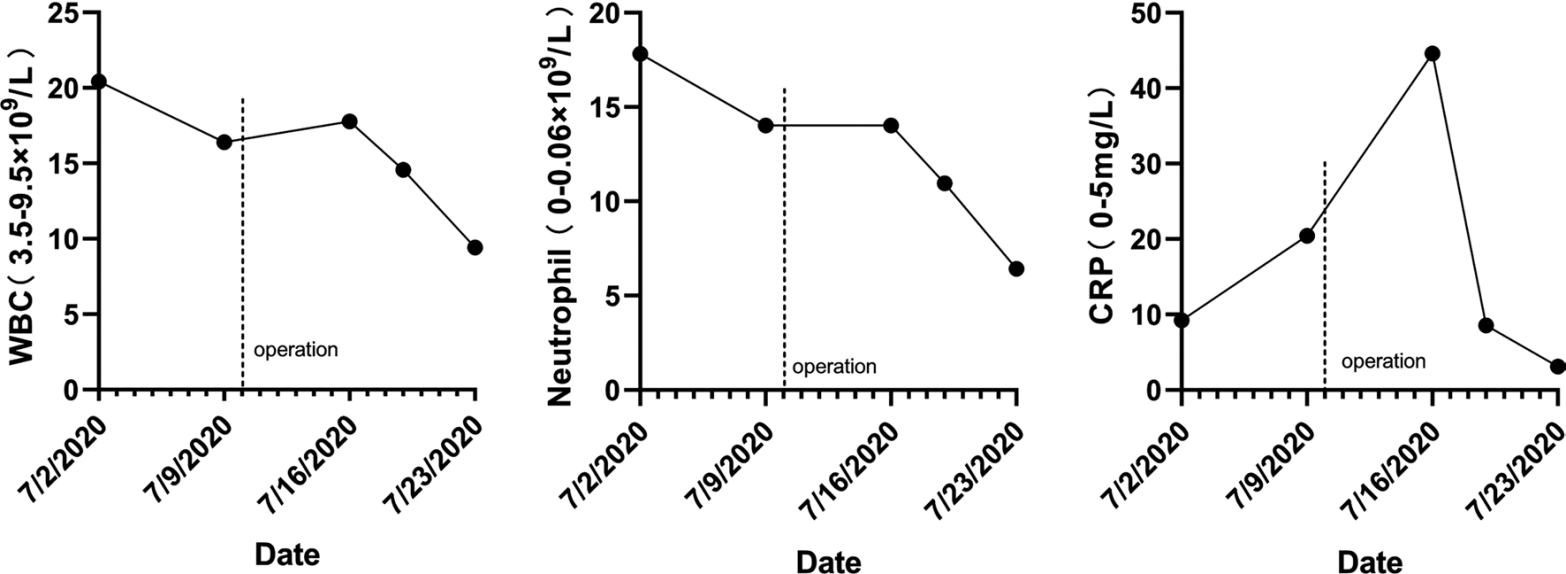
Changes of infection-related inflammatory indexes such as white blood cells (WBC), neutrophils and c-reactive protein (CRP) after surgery

Chest computed tomography (CT) scan was performed and showed multiple flocculent ground glass density shadows in both lungs after consultation with a respiratory physician, which could not be ruled out as viral pneumonitis. The patient had no epidemiological history related to coronavirus disease 2019 (COVID-19). COVID-19 was ruled out after the results of both oropharyngeal swabs and serum IgM/IgG for COVID-19 were negative. Meanwhile, test results of TORCH (TOX, RV, CMV, HSV), EB virus nucleic acid, and nine respiratory pathogens were all negative and the possibility of the patient suffering from viral pneumonitis was excluded. Meanwhile, she underwent a pulmonary function test that showed normal pulmonary ventilation, and the serum allergen-specific IgE test showed no seasonal allergy. Therefore, we considered preliminarily that pulmonary inflammation might result from chronic aspiration of CSF. The patient underwent endoscopic resection of meningoencephalocele and repair of skull base defect on July 10, 2020. We found a 4 * 5 mm^2^ bone defect in the lateral wall of the right sphenoid sinus (Fig. 2a), and a free mucosal flap of nasal floor was used to repair the defect. The patient’s dry cough disappeared on the first day after surgery. Pathological diagnosis revealed the resected lesion was brain tissue. Nasal endoscopy showed that the repaired area of the skull base defect healed well 2 months after surgery (Fig. 2b). Compared to preoperative imaging (Fig. 3a), pneumonitis seemed to be still in progressive stage with characteristics of the consolidation lesion with a mini cavity in the right lung 1 week after surgery although parts of multiple flocculent ground glass density shadows were gone (Fig. 3b). The lesion disappeared completely 2 months after surgery (Fig. 3c). Infection-related inflammatory indexes, such as c-reactive protein (CRP), white blood cells (WBC), and neutrophils were completely improved 1 week after surgery (Fig. 4).

## Discussion and conclusions

Aspiration pneumonitis accounts for 5–24% of community-acquired pneumonitis and about 5–15% of hospitalized pneumonitis [2, 3]. A meta-analysis showed that in-hospital and 30-day mortality was significantly increased in patients with aspiration pneumonitis compared to those with non-aspiration pneumonitis [4]. Therefore, it is very important to find out relevant etiological factors for aspiration in time for early diagnosis and treatment of aspiration pneumonitis.

The patient was not initially diagnosed with aspiration pneumonitis. One of the main reasons was similar clinical symptoms and chest CT imaging features with viral pneumonitis. Runny nose and dry cough, as common symptoms of respiratory tract infection, did not cause enough attention of the patient at the beginning. Most of the imaging findings of viral pneumonitis, including COVID-19, are manifested as multiple ground-glass density shadows in both lungs [5], which is similar to imaging features of aspiration pneumonitis. In addition, lack of identified etiological factors of aspiration was another major cause. We usually think that patients with esophageal diseases, neurological diseases or other chronic degenerative diseases are more likely to be exposed to the risk of aspiration. Moreover, the extent of exposure to persistent postnasal drip of CSF leading to macroaspiration is difficult to be witnessed directly in this case. These factors mentioned above delayed the awareness of etiological factor for aspiration pneumonitis.

Meningoencephalocele occurs mainly in infants with a very low incidence and is extremely rare in adults [6]. Zada et al. first reported a case about chronic aspiration-induced pneumonitis caused by spontaneous ethmoid sinus meningocele with CSF rhinorrhea, and indicated that increased diagnostic complexity associated with pulmonary complications led to unnecessary intervention and treatment delays [7]. CSF rhinorrhea can be considered as a cause of chronic pneumonitis despite the lack of direct evidence [8]. A controlled clinical trial has shown that sinonasal secretions can be transferred to the lungs whether in patients with cystic fibrosis or healthy subjects [9]. In this case, meningoencephalocele is located in the lateral wall of sphenoidal sinus and leads to persistent CSF rhinorrhea. Endoscopic surgery can be an effective and a safe treatment option for resection of meningoencephalocele and skull base reconstruction [10, 11]. A rapid improvement of symptoms, abnormal imaging findings and inflammation markers caused by pneumonitis after endoscopic surgery also proves that spontaneous sphenoid sinus meningoencephalocele with CSF rhinorrhea is a key factor to induce aspiration pneumonitis. However, the lesion could still progress further in the early postoperative period due to the persistence of the local inflammatory response caused by residence of components of CSF and the bacteria of nasal cavity and oropharynx along with CSF in the lungs. Continuous dry cough gives rise to raised intracranial pressure, which further increases flow of CSF and persistent aspiration. In addition, persistent cough caused by postnasal drip of CSF in the supine position may also cause reflux of gastric contents and increase the chance of aspiration. However, the direct evidence of aspiration pneumonitis caused by CSF rhinorrhea needs further clinical investigation.

Although uncommon, spontaneous sphenoid sinus meningoencephalocele with CSF rhinorrhea is an etiological factor for macroaspiration. Timely endoscopic lesion resection and repair of skull base defect can effectively avoid large-volume aspiration and make aspiration pneumonitis less likely to occur.

## Data Availability

The data that support the findings of this case are available from the corresponding author upon reasonable request.

## Abbreviations

CSF: Cerebrospinal fluid
CT: Computed tomography
COVID-19: Coronavirus disease 2019
CRP: C-reactive protein
WBC: White blood cells
